# Study on the Imaging Characteristics of β‐Amyloid (Aβ) Deposition (A) and Neurodegeneration (N) in Subjective Cognitive Decline

**DOI:** 10.1002/alz70856_104213

**Published:** 2025-12-25

**Authors:** Shuhua Ren, Yihui Guan, Fang Xie, Qi Huang

**Affiliations:** ^1^ Huashan Hospital, Shanghai, China; ^2^ Department of Nuclear Medicine and PET Center, Huashan Hospital, Fudan University, Shanghai, China; ^3^ Huashan Hospital, Fudan University, Shanghai, Shanghai, China; ^4^ Department of Nuclear Medicine & PET Center, Huashan Hospital, Fudan University, Shanghai, Shanghai, China

## Abstract

**Background:**

To investigate brain Aβ deposition, brain glucose metabolism, gray matter volume (GMV), and neurodegeneration features (e.g., hippocampus and parahippocampal gyrus volume), which are core pathological regions in Alzheimer's disease (AD), and to explore the correlation between these features and cognitive function in subjective cognitive decline (SCD).

**Method:**

This study included 144 cognitively unimpaired subjects from the Shanghai community: 95 SCD patients and 49 normal controls (NC). Participants underwent neuropsychological assessments, 18F‐Florbetapir PET/CT, 18F‐FDG PET/CT, and T1‐weighted MRI to evaluate cognitive status, Aβ deposition, brain glucose metabolism, GMV, and hippocampal/parahippocampal gyrus volume. The SCD group had self‐reported cognitive decline with concerns, while the NC group had normal neuropsychological tests and no Aβ deposition. Voxel‐wise two‐sample t‐tests and Mann‐Whitney U tests were performed to assess differences in brain Aβ deposition, glucose metabolism, GMV, and hippocampal/parahippocampal volume. Correlation analysis examined the relationship between these brain features and cognitive performance.

**Result:**

Compared to NC, SCD patients showed significantly higher Aβ deposition in the bilateral precuneus, posterior cingulate gyrus, and left frontoparietal lobe (*p* < 0.05). Mild brain atrophy was observed in the left occipital regions. SCD patients also had reduced glucose metabolism in several brain regions, including the right parahippocampal gyrus, while the left inferior parietal lobule showed increased glucose metabolism. Correlation analysis revealed that Aβ SUVr in SCD patients was positively correlated with executive function (STT‐A score), and hippocampal/parahippocampal volume correlated with overall cognitive, executive, and language functions.

**Conclusion:**

SCD patients exhibited specific Aβ deposition patterns, predominantly in the left hemisphere, with mild neurodegeneration. Aβ deposition correlated with executive function, while hippocampal/parahippocampal volume correlated with overall cognitive and language functions. These findings suggest potential imaging biomarkers for early AD diagnosis.

